# The History and Present State of Lunatic Asylums—Public and Private

**Published:** 1850-10-01

**Authors:** 


### THE JOURNAL

OF

### PSYCHOLOGICAL MEDICINE

AND

### MENTAL PATHOLOGY.

OCTOBER 1, 1850.

### Art. I.—THE HISTORY AND PRESENT STATE OF LUNATIC ASYLUMS—PUBLIC AND PRIVATE.

The history of lunatic asylums—their public and private administration—and the object and provisions of the present law of lunacy, are, we have reason to believe, very imperfectly understood; and although, upon account of our own peculiar position, we do not intend to enter into any controversy, or break a lance with every knight-errant who may choose to assail the present system of public and of private asylums, yet the subject is one which has a special and very serious claim upon our attention.

The ancients made no public provision, neither did they establish any hospital for the reception and treatment of the infirm and sick. In Athens, those who had suffered in the public service were fed in the *prytaneum;* but there was no asylum for them in case of sickness. In Sparta, where all the citizens assembled to eat together, there was no institution for the sick. The first establishment of hospitals is ascribed to the Christian era, when, about the end of the fourth century, Fabiola, a pious Roman lady, contemporary with St. Jerome and St. Ephraim, founded an institution for receiving the sick and the poor. Other hospitals were soon afterwards endowed. The Emperor Constantine built many; and the Emperor Julian did not hesitate to ascribe the progress of the Christian religion to these charitable institutions, and proposed, for the restoration of Paganism, that the example of the Christians should be imitated. We do not, however, meet with any accounts in ancient authors of hospitals appropriated to the insane. The first appears to have been established in the East. In the year 491, we read of one existing at Jerusalem. Benjamin of Tudela mentions that in the twelfth century, there was a large building at Bagdad, called *Dal Almeraphtan,* or House of Grace, in which those who lost their reason were received during the summer, and where they were kept in chains until they recovered. This house was visited by the magistrates every month, who examined the state of the patients, and released those who were well. In the same century, hospitals for the sick and the insane were founded by the Emperor Alexius. Among the Moors, by whom medicine was once diligently studied, hospitals and asylums for the insane were common. There was lately at Faz, or Fez, a *muriston* or mad-house, where the poor patients were chained down, and treated as cruelly as the lunatics were in the Timarahane at Constantinople, in the sixteenth century. Under the feudal system, in this country, when lands were held under military tenure, subject to military services, when the vassal was rendered incapable of performing such duties, the lord seized upon his rents and profits, and this custom applied to all infants and idiots. The lawless and violent practices of the ancient barons make it reasonable to suppose, that in consequence of the waste and spoliation that frequently took place of the property of persons labouring under mental imbecility and incapacity, the legislature was induced to place them under the immediate protection of the Crown; but the exact period at which this great change was effected is now unknown; nor can it be ascertained by what particular statute the king first acquired this jurisdiction. As Magna Charta makes no allusion to any prerogative of this nature, it seems to have had no existence in the time of John, and from the silence of Bracton, it was probably unknown in the reign of Henry the Third. Fleta informs us, that certain persons called *Tutores* used to have the custody of the lands *Idiotarum et Stultorum*, and he adds, that in consequence of an abuse of their trust, a statute was made in the reign of Edward I., by which the custody of the persons and inheritances *Idiotarum et Stultorum* being such *a nativitate*, was given to the king, with a reservation to the lord, of all his lawful claims for awards, relief, and the like. This statute, however, is not in print; but in the subsequent reign was passed the act "*De Prærogativa Regis*," the ninth chapter of which enacts, that "the king shall have the custody of the lands of natural fools, taking the profits of them without waste or destruction, and shall find them with necessaries, of whose fee soever the lands be so holden; and after the death of such idiots, he shall render it to the right heirs, so that such idiots shall not alien, nor their heir be disinherited." Also, "the king shall provide [habet providere] when any that before time hath had his wit and memory happen to fail of his wit; as there are many *per lucida intervalla*, that their lands and tenements shall be safely kept without waste and destruction, and that they and their household shall be maintained comfortably with the profits of the same; and the residue shall be kept for their use, to be delivered unto them when they come to be of right mind." Although Fleta mentions only persons who are afflicted with mental incapacity *a nativitate*, yet it is likely that some act, not now in print, prior to that mentioned by him of Edward I., gave the king jurisdiction in cases of lunacy also. This is Lord Rosslyn's opinion, who says that the words "habet providere" put it beyond all doubt; so that, in all probability, this jurisdiction was conferred upon the Crown in the early part of the reign of Edward I. Hence the duty of protecting those who are mentally incapable of taking care of themselves devolved originally upon the king in his capacity of *parens patriæ*, as a return for that allegiance which every subject owes him; and this is still the theory of the English law. To save the trouble and inconvenience of the sovereign personally adjudicating in every case, this duty is, by a special warrant, confided to the Lord Chancellor, who, by a recent act of parliament, is assisted by commissioners, and masters in lunacy; and in all places not within their immediate jurisdiction, by the justices of the peace in general or quarter-sessions. The lunatic, therefore, in reality, is "a state care," notwithstanding many who have written against the present law of lunacy appear to be ignorant of this fact. "The chancellor," observes Collinson, "acts by virtue of his general power as keeper of the king's conscience, and makes and enforces orders wholly foreign to any jurisdiction conferred by the king's signet." Hence an appeal lies from the chancellor to the King in Council, but not to the House of Lords.* So also, the "authority of the chancellor being personal, the Master of the Rolls cannot sit for him in lunacy." It should be added, that the right of the Crown to control and manage lunatics and their affairs begins with the verdict of an inquisition or commission.†

We may, from this brief sketch, perceive that the object of legislation was in the beginning to protect the person and the property of those who were incapable of taking care of themselves, little attention being paid to their medical or moral treatment; indeed, we have abundant evidence, that many who were so afflicted, were, before the foundation of lunatic asylums, treated with great cruelty. They were frequently thrown into prisons; and their hallucinations, from a misconception of their nature, punished with death. "Many of these poor creatures," says Reginald Scot, "had more need to be relieved than chastised; more mete were a preacher to admonish them than a jailer to keep them; and a physician more necessary to helpe them, than an executioner or tormentor to hang and burn them."‡ The oldest hospital in Europe for the

* A Treatise on the Law concerning Idiots, Lunatics, and other persons *non compotes mentis*. By George Dale Collinson. 2 vols. London, 1812.

† A Practical Treatise of the Law concerning Lunatics, Idiots, and Persons of Unsound Mind. By Leonard Shelford. 2nd edit. London, 1847.

‡ The Discoverie of Witchcraft. By Reginald Scot. London, 1665. Preface.

reception of the insane is Bethlem, or Bedlam. In the year 1247, in the 39th of Henry the Third, Simon Fitzman, who had been sheriff, influenced by the prevailing superstition of his age, was desirous of founding a religious house. Accordingly he appropriated, by a deed of gift which is still extant, all his lands in the parish of St. Botolph, being the spot known by the name of Old Bethlem, to the foundation of a priory. The prior, canons, brethren and sisters, for whose maintenance he provided, were distinguished by a star upon their mantles, and were especially directed to receive and entertain the Bishop of St. Mary of Bethlehem, and the canons, brothers, and messengers of that order, as often as they might come to England. Such was the original design of this foundation. During the succeeding 200 years, we hear little more of this house; until Henry VIII., in abolishing monasteries, seized upon it, and in the year 1547, granted it as the hospital of Bethlem, with all its revenues, to the mayor, commonalty, and city of London, from which time it became an hospital for the cure of lunatics. Insanity, owing doubtless to the exciting commotions upon religion, was at that period a prevalent malady. Hence, Bowen observes,—"It is most probable that the city of London had felt great inconvenience for the want of a proper receptacle for those unhappy objects who were afflicted with the most deplorable malady incident to the human frame. The retired situation of the hospital of Bethlem, and its contiguity to the city, pointed it out as a fit place for the desired object. Accordingly, we find, from authentic documents, that in the year 1523, Stephen Gennings, Merchant Taylor, gave 40*l.* by will towards the purchase of this hospital, and that the mayor and commonalty had taken some steps to procure it, a very short time before they derived their right to it from royal munificence."*

That the insane continued to increase in numbers, or that more attention was paid to the disease, is obvious from the fact, that it became necessary to build a new and larger hospital for their reception. In the year 1644, it was proposed to enlarge the old building; "but the situation," says Bowen, "was too close and confined to allow of its being rendered a commodious asylum for the numerous distracted persons of both sexes who claimed its protection, and probably the dreadful commotions of that period checked the idea of improvement. When legal government was restored, and England had rest from the violence with which it had been convulsed, the concerns of civilized society were again attended to, and it became a matter of serious deliberation to build a new hospital. In April, 1675, this great work was commenced. The lord mayor, aldermen, and common council of the city, allotted to

* An Historical Account of the Origin, Progress, and Present State of Bethlem, founded by Henry VIII. By the Rev. Thomas Bowen. London, 1783.

the governors a large piece of ground, near London-wall, on the south side of the lower quarter of Moor-fields, where Bethlem now stands. The expedition with which this stately edifice was completed challenges our admiration; for from an inscription over the arch facing the entrance into the hospital, it appears that it was finished in the July of the year following. The expense of the building amounted to 17,000*l*."* A curious circumstance is mentioned. The design of the building was taken from the Tuilleries in Paris; and it is said that Louis XIV. was so much offended that his palace should be taken for a model of a lunatic asylum, that, in revenge, he ordered a plan of St. James's to be taken, for offices of a very inferior description.† There were formerly two remarkable looking figures over the gates of the hospital, which will now be found in the entrance-hall of the present building,—two stone figures, representing Mania and Melancholia, which were the work of Cibber, the father of the comedian. "My father," says Colley Cibber, "whose name was Caius Gabriel Cibber, was a native of Holstein, and came to England some time before the restoration of King Charles II. to follow his profession, which was that of a statuary. The basso of the pedestal of the great column in the city, and the two figures of the lunatics, the raving and the melancholy, over the gates of the Bethlem Hospital, are no ill monument of his fame as an artist."‡

When this hospital was erected, it was hoped that adequate provision would be found for all lunatics who were dangerous to the public; but we are told that "so great was the influx of insane persons from all parts of the kingdom," that it was again found necessary to enlarge the building, and, in the year 1734, two additional wings, capable of receiving 100 incurables of each sex, were added. In the year 1751, Bethlem being insufficient in size to admit the numerous cases which presented themselves, St. Luke's was founded by voluntary subscription; and the example of London was now followed by Manchester, York, and other large cities. The first asylum in France for the reception of lunatics was founded at Marseilles in the year 1600. In the year 1657, there were eight incurable lunatics in the Petites Maisons of Paris;—and throughout the provinces the insane were indiscriminately confined in religious houses and prisons. The rich and the poor in Paris were sent to the Hotel Dieu, where two large rooms on the first floor were set apart for males and females; and here lunatics, without any classification, and even hydrophobic patients, were confined. To the philanthropy and zeal of M. Tenon, the French are indebted for those

* An Historical Account of the Origin, Progress, and Present State of Bethlem, founded by Henry VIII. By the Rev. Thomas Bowen. London, 1783.

† We do not know where Bowen found this anecdote.—*Ed.*

‡ Colley Cibber's Apology for his Life. London, 1780.

noble establishments for the insane which are justly now the admiration of Europe. It appears that M. Tenon, after visiting Bethlem, and other lunatic asylums in England, submitted a memoir to the French Government in 1786, proposing to withdraw the insane from the Hotel Dieu, and place them in asylums specially erected for their reception. In 1787, M. Soulaire, who had come to England for the purpose of making some observations in natural history, visited Bethlem, and was so struck with the expediency of public asylums, that on his return to Paris, he published a pamphlet on the origin, progress, and state of Bethlem, which created considerable sensation. In all the principal provinces of France, and throughout Europe, lunatic asylums were now established, and placed under the supervision of the different governments and local authorities. Private asylums at the same time sprung into existence, for the relations and friends of the rich very naturally refused to place the members of their afflicted families in the asylums of the poor, or derive support from public charity. Another feeling, which is deeply implanted in human nature, also appears to have necessitated these private establishments—viz., the desire of drawing a veil over family misfortune, and avoiding the exposure of so cruel an affliction to the world.

The establishment of public and private lunatic asylums has been attended with many very obvious advantages, which we should scarcely consider necessary to point out, were there not so much misunderstanding and prejudice abroad on this subject. The inhumanity and cruel treatment which lunatics formerly experienced, in those ages when the most palpable hallucinations were considered treason and blasphemy, we have already alluded to. We read of three unhappy lunatics in the reign of Queen Elizabeth, Arthington, Coppenger, and Hacket—the first of whom was hanged, drawn, and quartered—the second imprisoned, and died raving—the third, upon recovering his senses, was pardoned. The offence of Arthington was, that he believed Coppenger to be a prophet of mercy, and Hacket king of Europe, who were to go before him, and separate the sheep from the goats.* Another lunatic, named Venner, being under the delusion that all human government was about to cease, proclaimed our Saviour king, in the public streets. He was followed by a rabble, who were attacked by the militia, and taken prisoners. Poor Venner was executed January, 1660, protesting his belief that Cromwell and Charles II. were Christ's usurpers, and twelve of his followers, under the same delusion, shared the same fate.† The porter of Oliver Cromwell, named Daniel, met with more gentle treatment. He became deranged from poring over mystical books of

* Biographia Britannica. Vol. i. Art. "Arthington." London, 1774.
† Granger's Biographical History of England. Vol. iv. p. 208. London, 1784.

divinity, and was for many years confined in Bethlem, from one of the windows of which he would frequently preach, chiefly to females, who would often sit for many hours under his window very busy with their bibles turning to the texts he quoted.*

The prejudice against, or rather the inhuman feeling, entertained towards persons mentally affected, is curiously illustrated by an anecdote mentioned in the History of the Royal Society, recently published by Mr. Weld. When experiments were being made, in 1660, upon the transfusion of blood, "an account was received by the society of two experiments made in Paris before the Academy of Sciences on a youth, and on an adult, whose veins were opened, and injected with the blood of lambs. The experiment, according to the account, succeeded so well, that the Society became anxious to perform it upon an individual, and Sir George Ent suggested that 'it would be most advisable to try it upon some mad person at Bethlem.' This proposal met with the general approbation of the Royal Society, and a committee was forthwith appointed to communicate with Dr. Allen, the physician, and to request him to furnish a lunatic for the experiment." It is somewhat satisfactory to find that Dr. Allen declined acceding to this request; and the committee reported "that Dr. Allen scrupled to try the experiment on any of the mad people at Bethlem." They were then ordered to consider together how the experiment might be most conveniently and most safely tried.† In Paris, the same operation was attempted by M. Denis and le Seur Emerez, and the poor lunatic, during the process, died in their arms. We have elsewhere alluded to the superstitious feeling and dread which even educated and professional men have occasionally betrayed in approaching the insane; and we have further evidence of this popular prejudice in the very lively letters of Horace Walpole to the Countess of Ossory. "One project," says Horace Walpole during the Gordon Riots, "of the diabolical incendiaries, was to let loose the lions in the Tower, and the lunatics in Bethlem. The latter," he adds, "might be from a fellow-feeling with Lord George; but cannibals do not invite wild beasts to their banquets."‡

In the early history of lunatic asylums, whether we look into the interior of Bethlem or the Bicêtre, we must not be surprised to find a want of remedial treatment and moral management towards the inmates, because at this period the nature of the disease was little understood, and the primary object was considered, very naturally, to be the

* Granger's Biographical History of England. Vol. iv., p. 208. London, 1784.

† History of the Royal Society. By Charles Richard Weld, Esq. London, 1848.

‡ Letters addressed to the Countess of Ossory, from the year 1769 to 1797. By Horace Walpole Lord Orford. 2 vols. London, 1848. Letter clxxii. vol. i. p. 441.

safe custody of the lunatic. But we must not confound the past with the present;—the interior aspect of a lunatic asylum a century or two ago bears no resemblance whatever to the interior of asylums—public or private—as they are now conducted. Take for example Hogarth's celebrated picture, the "Scene in a Mad-house;" the culminating point of retribution which awaited the Rake's progress is here given with consummate skill, and we behold the interior of Bethlem, not as it now exists, but as the imagination of the artist beheld it when no attention was paid to classification, and instruments of coercion were the only means adopted to restrain violence. The hero of the piece is there seen chained by the leg, lying naked on the ground, tearing himself, in a state of fury, to pieces; while he is supported by the unhappy female whom he cruelly betrayed, but who still followed him through all the vicissitudes of his evil fortune. Near to him is a poor lunatic gazing through a roll of paper, as if through a telescope; and before him, a crazy tailor playing with his measure and looking wildly at the mad astronomer, wondering, through excess of ignorance, what discoveries the heavens can possibly afford. Upon one side we observe another, who imagines himself the pope, and is saying mass in a pompous style; and opposite him another, with his head encircled with a straw crown of royalty, who fancies himself "every inch a king." The figures are all of them painfully true to nature; and in the midst of this dreary scene may be observed two gaily-dressed ladies, brought thither by an idle curiosity to gaze on the deplorable spectacle around them. We are informed in the History of Bethlem "that the hospital used formerly to receive a revenue of 400*l.* per annum from the indiscriminate admission of visitors; but this liberty, though beneficial to the funds of the charity, was thought to counteract its grand design, as it tended to disturb the tranquillity of the patients; it was therefore judged proper, in the year 1770, no longer to exhibit the house to public view, except by special order."* That very accomplished and elegant writer, Henry Mackenzie, has in the "Man of Feeling" given a very touching picture of the interior of Bethlem:—

"Of those things called 'Sights in London,' he observes, "which every stranger is supposed desirous to see, Bethlem is one. To that place, therefore, an acquaintance of Harley's, after having accompanied him to several other shows, proposed a visit. Harley objected to it, 'because,' said he, 'I think it an inhuman practice to expose the greatest misery with which our nature is afflicted to every idle visitant who can afford a trifling perquisite to the keeper, especially as it is a distress which the humane must see with the painful reflection that it is not in their power to alleviate it.' He was overpowered, however, by the soli-

* Bowen. Op. cit.

citations of his friend and the other persons of the party, (amongst whom were several ladies,) and they went in a body to Moor-fields. Their conductor led them first to the dismal mansions of those who are in the most horrid state of incurable madness. The clanking of chains, the wildness of their cries, and the imprecations which some of them uttered, formed a scene inexpressibly shocking. Harley and his companions, especially the female part of them, begged their guide to return. He seemed surprised at their uneasiness, and was with difficulty prevailed upon to leave that part of the house without showing them some others, 'who,' as he expressed it, in the phrase of those that keep wild beasts for show, 'were much better worth seeing than any they had passed, being ten times more fierce and unmanageable.' He led them next to that quarter where they reside who, as they are not dangerous to themselves or others, enjoy a certain degree of freedom according to the state of their distemper. Harley had fallen behind his companions, looking at a man who was making pendulums with bits of thread and little balls of clay. He had delineated a segment of a circle on the wall with chalk, and marked their different vibrations by intersecting it with crossed lines. A decent looking man came up, and smiling at the maniac, turned to Harley, and told him that gentleman had once been a very celebrated mathematician. 'He fell a sacrifice,' he said, 'to the theory of comets; for having, with infinite labour, formed a table on the conjectures of Sir Isaac Newton, he was disappointed in the return of one of those luminaries, and was very soon after obliged to be placed here by his friends.'"

The keeper points out to Harley other patients, labouring under different and remarkable delusions, and the visit to Bethlem concludes with one of the most beautiful and pathetic descriptions in the English language:—

"Separate from the rest stood one whose appearance had something of superior dignity. Her face, though pale and wasted, was less squalid than those of others, and showed a dejection of that decent kind which moves our pity unmixed with horror; upon her, therefore, the eyes of all were immediately turned. The keeper who accompanied them observed it. 'This,' said he, 'was a young lady who was born to ride in her coach-and-six. She was beloved, if the story I have heard be true, by a young gentleman, her equal in birth, though by no means her match in fortune; but love, they say, is blind, and so she fancied him as much as he did her. Her father, it seems, would not hear of their marriage, and threatened to turn them out of doors if ever she saw him again. Upon this, the young gentleman took a voyage to the West Indies, in the hopes of bettering his fortune and obtaining his mistress, but he was scarce landed, when he was seized with one of the fevers which are common in those islands, and died in a few days, lamented by every one that knew him. This news soon reached his mistress, who was at the same time pressed by her father to marry a rich, miserly fellow, who was old enough to be her grandfather. The death of her lover had no effect on her inhuman parent; he was only the more earnest for her marriage with the man he had provided for her; and what between her despair at the death of the one, and her aversion to the other, the poor young lady was reduced to the condition you see her in. But God would not prosper such cruelty; her father's affairs soon afterwards went to wreck, and he died almost a beggar.' Though this story was told in very plain language, it had particularly attracted Harley's notice; he had given it the tribute of some tears. The unfortunate young lady had till now seemed entranced in thought, with her eyes fixed on a little garnet ring she wore on her finger: she turned them now upon Harley—'My Billy is no more,' said she. 'Do you weep for my Billy? blessings on your tears! I would weep, too, but my brain is dry, and it burns—it burns—it burns!' She drew near to Harley. 'Be comforted, young lady,' said he, 'your Billy is in heaven!' 'Is he, indeed! and shall we meet again? Alas! I am grown haughty of late; I have almost forgotten to think of heaven: yet, I pray sometimes—when I can, I pray; and sometimes I sing; when I am saddest, I sing—you shall hear me—hush!

"Light be the earth on Billy's breast,
And green the sod that wraps his grave."

There was a plaintive wildness in the air not to be withstood; and except the keeper, there was not an unmoistened eye around her. 'Do you weep again?' said she; 'I would not have *you* weep. You are like him, believe me; just so he looked when he gave me this ring. Poor Billy! 'Twas the last time ever we met—

"'Twas when the seas were roaring."

I love you for resembling my Billy, but I shall never love any man like him.' She stretched out her hand to Harley: he pressed it between both of his and bathed it with his tears. 'Nay, that is Billy's ring,' said she; 'you cannot have it, indeed; but here is another—look, here, which I plaited to-day, of some gold thread from this bit of stuff: will you keep it for my sake? I am a strange girl, but my heart is harmless; my poor heart, it will burst some day—feel how it beats.' She pressed his hand to her bosom, then holding her head in the attitude of listening—'Hark! one! two! three! Be quiet, thou little trembler! My Billy is cold! But I had forgotten the ring.' She put it on his finger—'Farewell! I must leave you now.' She would have withdrawn her hand—Harley held it to his lips. 'I dare not stay longer; my head throbs sadly—farewell!' She walked with a hurried step to a little apartment at some distance. Harley stood fixed in astonishment and pity; his friend gave money to the keeper. Harley looked on his ring. He put a couple of guineas into the man's hand—'Be kind to that unfortunate.' He burst into tears, and left them."*

The exquisite simplicity and pathos of this story will apologize for the digression. To return. The state of lunatic asylums not only in England, but in France, Italy, and Germany, until their organization

* The Man of Feeling. By Henry Mackenzie. Chap. xx. Edinburgh, 1808.

and management became better understood, was very deplorable. When Esquirol, some thirty years ago, visited the asylums in France, he found them literally dungeons of wretchedness. When he visited the Salpêtrière, he expressed his horror at perceiving one of the unfortunate patients lying, in a state of nudity, on the bare ground, with scarcely sufficient straw to cover him. The attendant, observing his astonishment, immediately explained that he was only allowed a small quantity of straw to distribute every second day. "What!" exclaimed the indignant physician, "the dog at the threshold of the building is better housed." The use of chains in restraining lunatics is extremely ancient. They were often rivetted to the body, and used generally throughout Europe, until the year 1794. No other method of controlling a violent patient was then dreamed of; but, happily for the cause of humanity, Pinel determined to liberate the poor lunatic from his fetters, and unchained, in a single morning, at the Bicêtre, as many as eighty patients, not one of whom, when set free, committed a single act of violence. This noble example was followed by all the principal asylums in Europe. In Italy, however, the progress of this reformation in the management of lunatic asylums was slow. We are informed by Lee, in his "Observations upon Medical Institutions," upon the authority of Brierre de Boismont, that in Genoa, the Hospital for the Insane was, so recently as 1835, in the following wretched condition:—"The women are shut up in two dirty and badly-lighted wards: one of them is large, damp, and cold. Many of the patients are chained by the hands and feet; their howlings, their accessions of fury, and the clanking of their chains, give to this horrible place the appearance of the infernal regions."* He informs us, also, that chains were still used in the Hospital for the Insane at Rome.

"In several of the courts and corridors, chains with a ring for the neck or foot are fixed to the walls to confine furious patients. Some of these patients occupy cells in the courtyard; the others sleep in small but clean wards, containing each from ten to twelve beds. There is no division of the varieties of insanity, but all are mixed indiscriminately together. The strait-waistcoat is the usual means of restraint, chains being only used in extreme cases. No moral measures are resorted to in the treatment, which seems to consist principally in bleeding, warm, cold, and douche baths. The number of cures I should suppose to be very small. At Aversa, near Naples, however, is one of the best hospitals for the insane in Italy, which was organized by Professor Vulpez. Chains are here dispensed with. At one end of a gallery is a small chamber, containing several vertical beds for furious patients, whose legs are placed in a kind of case, lined with leather, and fastened by a padlock; the strait-waistcoat is also used."†

* Observations on Medical Institutions in France, Italy, and Germany. By Edwin Lee. London, 1835.

† Ibid.

It should be added that insanity is less common in Italy than in France, England, Scotland, or Norway.

The amelioration in the condition and management of lunatic asylums in England may be dated from the years 1815—1816; when a select committee was appointed to report on the state of Bethlem, and consider of provisions being made for the better regulation of mad houses. The publication of the reports, which followed, excited an intense interest and excitement throughout the country, and physicians connected with public asylums and the proprietors of private asylums emulated each other in endeavouring to improve the condition and management of their establishments. Several acts of parliament were now successively passed, with the view of ensuring the proper government of these establishments—but as these are now rescinded, the history of the provisions contained in these statutes ceases to be of importance. The act under which lunatic asylums are now governed is the 8th and 9th Vict., cap. 100, under which the Lord Chancellor, officiating for the sovereign as the *parens patriæ*, upon the principle we have above explained, is authorized to appoint Masters in Lunacy and Commissioners, whose duties are severally specified in the different sections of the act. The commissioners are empowered to grant licences for asylums, without which licence no house for the reception of more than two lunatics can be opened. They have, then, an immediate jurisdiction delegated to them over the management of all these establishments, and are required to visit them personally, in order to satisfy themselves that the patients are properly and humanely treated. The public, we apprehend, would not fail to have every confidence in these establishments if the jurisdiction of the commissioners over them were better understood. We may observe, by referring to the reports addressed to the Lord Chancellor, the principles upon which the commissioners proceed in granting and revoking licences; in sanctioning the admission, or ordering the discharge of patients, in correcting incidental abuses, whether in respect to medical or moral treatment, restraint, clothing, bedding, diet, &c. It will be found that they listen readily to any complaints which may be made respecting the management of patients, and institute special investigations in cases where charges of a serious nature are preferred against the parties managing asylums, or against the parties who may be accused of having unjustly caused the detention of any patient. The ever-constant surveillance of the commissioners affords, we repeat, a security to the public which we feel assured would be esteemed all-sufficient, were the nature of the duties which they discharge only properly appreciated.

In order to insure the comfort of patients on their admission into private lunatic asylums, the commissioners adopt the following precautions in granting licences :—On receiving an application to open an asylum, they appoint two members of the commission to inspect the house proposed to be licensed ; and if they report it to be unobjectionable in point of structure, and to be sufficiently spacious, and otherwise fit for the number of patients proposed, they intimate to the applicant their intention to license the house, if on a second inspection it be found properly furnished. They, however, do not grant the licence until the house is actually in a fit and proper state for the reception of the patients. More than this : before entrusting any person with a licence to receive insane patients, they require testimonials of moral character and professional skill and experience ; and they require also the applicant to show that he possesses pecuniary means for enabling him to carry on and maintain the establishment in a comfortable state. They have also, in case of being dissatisfied with the conduct of a proprietor, full power to revoke the licence, which is done by the Lord Chancellor, under their recommendation.

The commissioners in lunacy are extremely particular that the orders and medical certificates for the admission of patients into asylums shall be exactly in the form prescribed by the act; and here we at once come to the subject-matter of much dispute. Are the forms at present prescribed for the admission of a patient into an asylum sufficient? Is the liberty of the subject liable to be in any way unjustly assailed upon the plea of insanity? We have already upon this point expressed our conviction that to obtain the incarceration of a sane person upon such an allegation in any private asylum would imply an extent of confederacy and a combination of collateral circumstances which we do not apprehend could possibly occur. We require, before any such admission be made, the order of reception to be signed by some member of the family, or friend, who is in the first instance a responsible party ; we next require certificates from two medical men, who are unconnected with each other, and who have each separately examined the patient; we next find that the medical proprietor, or superintendent of the asylum, is required to forward a notice of the admission of the patient to the commissioners, specifying what may be his or her mental state and bodily condition; lastly, we have the supervision of the medical section of the commission. We cannot conceive parties so unconnected as those with each other, and so absolutely disinterested in such a matter, conspiring, and that successfully, together to deprive a fellow-subject of his liberty without any ostensible motive for doing so. Besides, it behoves those who consider these forms insufficient, to suggest some better and more guarded plan in their place, for there can be no doubt that insanity, as a very frightful disease, does exist, and that persons so affected require to be taken care of. There has been for some years past a society in

London, called the "Alleged Lunatic Friend's Society," and the gentlemen who belong to it amuse themselves in writing pamphlets and newspaper paragraphs, and in holding occasional public meetings to expostulate among themselves against the present law of lunacy; but we have not yet heard of their suggesting any amendment or remedy for the evils and abuses which they allege exist. It was certainly proposed by the Amendments Law Society that the verdict of a jury should be substituted for the present order of admission and the two medical certificates; but we are satisfied that such a plan would be found wholly impracticable. We do not believe that any family suffering under such a calamity would willingly permit a relative so afflicted to pass through the exposure of such an ordeal. It should be remembered that for many cogent reasons the sanctity of the domestic circle should, under such distressing circumstances, be held inviolate.

"Those who are unacquainted," observes Dr. Forbes, "with the history of families over which the plague of insanity has fallen, know but a part of the miseries incident to human beings. If they could behold the accumulated trials of wives, daughters, and mothers under such circumstances; the immediate privations, the alarm and agitation, the sacrifice long endured for those who repay such devotion with frantic abuse, with an ingratitude, the result of disease, but which does not the less wound and grieve the hearts of those who still love the doomed and falling creature, whose sense and whose character are alike undergoing ruin; they would be convinced that there is no sorrow like their sorrow."*

We affirm, then, that to expose a family so afflicted to the examinations and cross-examinations of a petty trial by a jury would be abhorrent to the most sacred feelings of humanity. Besides, we are by no means satisfied that the verdicts of juries generally in such cases are so clear and conclusive as the expressed judgment of medical men who understand the phenomena of the disease upon which they adjudicate. The popular notion is, that a lunatic must be incoherent and rampant; he must rave, and tear his clothes; he must appear to be dangerous to himself, or dangerous to others, by attempting to perpetrate acts of insane violence; he must resemble one or other of the lunatics in Hogarth's "Scene in a Mad House," or in Henry Mackenzie's description of Bethlem, or the popular opinion will not recognise any evidence of positive mental aberration—albeit, at that moment, the malady may be progressing, and the patient still in a curable condition. In France, the government requires the certificate only of a single physician,† and, in cases of emergency, even that is dispensed with; but we

* The British and Foreign Review. Vol. ix. p. 187. London, 1840.
† Loi sur les Aliénés. Sect. i. art. 8, § 2.

are confident that the English public would not be satisfied with the custody of the insane being committed to the order of the prefect of the police, or any other individual official authority. The immediate relations, two physicians, or general practitioners independent of one another, the report of the medical proprietor, or superintendent of the asylum, and the concurrent sanction of the commissioners in lunacy comprehend, in our estimation, a far more competent and satisfactory tribunal than any other which we have yet heard of.

Under the system now established, let us look fairly at the improvements which have taken place in public and private asylums.

"It is admitted by all persons well acquainted with the subject," observe the commissioners,* "that the receptacles for the insane have undergone great although gradual improvement during the last few years. That this has been owing in a considerable degree to the public attention having been lately more directed to them, and to the treatment of insanity being now better understood than formerly, there can be no doubt. At the same time, we are satisfied that the good condition of these establishments, more especially of the licensed houses [private asylums], is mainly owing to the special supervision to which they are constantly subject; and it would not be difficult, we think, to trace a very large proportion of the improvements which have taken place in various asylums and houses receiving lunatics to the suggestions of the persons [commissioners, committees of visitors, and visiting magistrates] under whose supervision they have from time to time been placed."

Let us here also request attention to the following official statement, which we adduce as an irrefragable argument in favour of the present administration of the law of lunacy; and we think that it ought to—nay, that it will—have its due weight with the public.

"Without adverting," the commissioners observe, "to the many cases where persons have been restored to the world by means of such intervention, important benefits and comforts of various sorts have been obtained for insane patients by the present system of inspection and supervision. The dwellings for the insane are no longer the gloomy prisons in which they were formerly confined; cleanliness, warmth, and ventilation have been provided; personal restraint is diminished, and even where still employed, its severity is greatly mitigated, and its application strictly watched; the health and mental condition of the lunatic are more carefully considered, occupation and amusement are more generally afforded to him; and in all respects better treatment is secured; whilst an opportunity is periodically given to him of representing any hardship to which he may have been subjected, an advantage which it is found by experience, many patients fully appreciate."†

* Further Report of the Commissioners in Lunacy. 1847. p. 61.
† Ibid., p. 12.

We might select from the numerous reports of the county asylums which lie upon our table the most conclusive evidence that the age of darkness has passed away, and that public and private lunatic asylums generally throughout the country are conducted upon the most enlightened and humane principles. But we must not expect to find an Utopia of happiness and contentment in those establishments, for the very nature of this distressing malady produces restlessness, irritation, and an almost incurable dissatisfaction with every comfort that life itself can afford. The complaints, therefore, of patients—and of men who have been patients—should be received with very great caution. In the last report of the Crichton Royal Institution, Dr. W. A. F. Browne very truly remarks that—"Besides the obvious and legitimate ground for suffering, there is at all times a large amount of discontent among the insane, which does not necessarily flow from their delusions, or from the restrictions of seclusion, and which it is difficult to combat or allay by appeals to patience and resignation, or by the distractions of occupation and recreation. There are spirits who court strife, who may disregard sympathy, but who delight in publishing their woes. Self-tormentors never fail to torment others, or to diffuse the querulous, captious, and dissatisfied tone of their own mind to all around."* In particular forms of insanity, such as the *manie raisonnante*, for example, of Esquirol, this morbid state of irritability is very remarkable, and will continue for years after a patient is discharged, professedly cured. Dr. Browne, in continuation, observes—"It has been said, sneeringly but truthfully, that not a tenth of those who display satisfaction with their abode would continue inmates in an asylum were liberty unconditionally offered. Escapes are comparatively rare, but the desire for freedom, for the unlicensed exercise of the will, is intense; wandering is in itself a morbid manifestation, and it is accordingly found that patients are ready and anxious to exchange present comfort for prospective anxieties and difficulties, and the power to go from place to place."† Notwithstanding this state of morbid restlessness, so well described by Dr. Browne, patients in their happier and healthier moments, after their liberation, view the asylum in which they have been confined and kindly treated with very different feelings. "It is well known," says the late Dr. Millingen, "that patients on their discharge from an asylum, upon the approach of a fresh attack, have come to request a readmission; a convincing proof that they do not look upon these establishments with the horror they are generally supposed to entertain of them."‡

* Tenth Annual Report of the Crichton Royal Institution for Lunatics. Dumfries, 1850.

† Aphorisms on the Treatment and Management of the Insane. By J. G. Millingen. London, 1840.

‡ Ibid.

The Commissioners in Lunacy appear to be desirous that restraint should be entirely abolished; but cases occur occasionally in which they acknowledge its expediency. Following the example of Pinel, Dr. Charlesworth has the merit of being the first in England who dispensed with every form of coercion at Lincoln Asylum, and all his views respecting the medical and moral treatment of the insane are characterized by the greatest humanity and liberality. Every form of restraint was next abolished by Mr. Tuke, in The Retreat, near York, and afterwards by the Hanwell Asylum; but the subject is one which is still *sub judice.* The substitution of human for mechanical restraint under some circumstances adds very much to the excitement of an infuriated patient, and even the padded room in many cases is by no means to be commended. This description of safety-room was, it would appear, invented by the late Dr. Autenreith, "who, to obviate the necessity of bodily restraint by the ordinary means," says Dr. Burrows, "constructed a strong room, padded all round, in which he conceived the most furious lunatic might be let loose, like a beast in a den, without doing harm to himself or to any one. The absurdity and uselessness of such a plan," continues Dr. Burrows, "must be apparent to the experienced, who know that some maniacs unrestrained and so situated would tear away all padding, and beat their brains out, or soon become beasts in reality."* We have seen many cases of mania complicated with epilepsy in which no doubt, from inflammation or irritation at the base of the brain, patients have been inclined to throw and beat themselves about so violently as to give a concussion to the system which cannot fail to be very injurious to them, and even the padded walls in such rooms as we find at Hanwell are sufficiently firm to give an amount of resistance sufficient to augment the shock. Nevertheless, there are many cases in which the padded room, particularly when darkened, may be used with great advantage, and produce a tranquillizing and beneficial effect. There can be no doubt—and it is gratifying to find that there has been in all asylums—a gradual diminution, amounting almost to a positive disuse of every form of restraint. The absolute necessity of exerting, if not a physical, a moral control over violent patients may, in truth, be adduced as a very strong argument in favour of the general management of asylums, for the clumsy manner in which every endeavour to effect restraint is attempted and rarely with success, at home, goads patients into a state of the highest frenzy. In the former report of the Commissioners, they state that mechanical restraint generally is

* "Those who wished," adds Dr. Burrows, "to read Autenreith's description of this room, and how to manage violent and obstinate patients, must consult the Clinical Annals of Tubingen, [vol. i. part i. 1807,] but I fear the perusal will scarcely compensate for the trouble." Commentaries on the Causes, Forms, and Treatment of Insanity. By George Man Burrows, M.D. London, 1828. p. 689.

diminished, and that in licensed or private asylums they find "the practice of coercion is an exception to the general rule of treatment. The massive bars, and rings, and chains of iron, formerly resorted to, are no longer seen. Long-continued coercion is not permitted, and coercion itself scarcely ever allowed, except with the sanction of the medical officer, who is himself compelled by the act of parliament to record every week, in a journal kept framed for the purpose, the name of every patient under restraint and in seclusion, the duration of the restraint—whether it be only for a single hour or even a few minutes—and the means by which such restraint is effected." Here, then, as affects the question of restraint, every protection is thrown around the patient within the asylum that can well be devised.

The commissioners, at their several visitations, see every patient in the asylum, and grant private interviews to those who may have any grievance to complain of, or who may otherwise wish a conference with them. They visit the day-rooms, and night-rooms, and every part of the establishment, and examine the bed and bed-clothes, with the view of ascertaining their condition in respect to cleanliness and quantity. They inspect the quality of the different articles of diet, and extend their inquiries generally, to every subject connected with domestic comfort. What more can be desired? Surely the opponents of the present system would not wish the doors of either public or private lunatic asylums to be thrown open to the inspection of the public generally, for the gratification of the idle and the curious, as we have seen was permitted when Bethlem was in its unreformed and worst condition. The relations and friends—no matter who they may be—if the proprietors or superintendent conceive it right to refuse their seeing a patient, can always, if their applications be proper, obtain an order for this purpose from the commissioners, which must be obeyed. There is no concealment, therefore, of any person so confined contemplated or permitted by the act; but there may be many cogent reasons for refusing certain parties access to particular patients; and in this matter the proprietors must be guided by the instructions they receive from the more immediate relations—else, under peculiar circumstances, they must be guided by the advice of the commissioners. But it may be argued that certain cases have been tried in our public courts which have exposed a very great amount of abuse to have taken place in some establishments. We readily admit the fact, which we contend proves the efficiency of the surveillance exercised by the commissioners. The transgression of a law and the conviction of the transgressor for violating that law, is no proof of the inefficiency of the law itself; and were the names of the parties made known who have been arraigned before the tribunal of the commissioners for various irregularities arising from mistakes in the forms of the returns and reports required by the act, and for other, graver or minor, offences, the vigilance of the commissioners would, we are satisfied, be duly appreciated, and the present system of conducting these asylums clearly vindicated.

The general improvement which has taken place in lunatic asylums throughout the kingdom will not fail to be manifest to all who will give themselves any trouble to examine the subject candidly. An active and able medical superintendence is now established in every asylum, and the principle acknowledged that these institutions are intended not so much for the safe custody of patients, however important that object may be, as for immediate and active medical treatment. This is the primary object, and the cures effected in them alone prove the advantages which they command, for it is notorious that patients recover in asylums who would become incurables at home. Besides medical advice, these establishments command moral resources which materially assist the remedial treatment. Exercise, occupation, amusements are provided to withdraw the mind from its cloud of morbid associations. Every well-appointed asylum is provided with a billiard-table, bagatelle boards, books—often an extensive library; and the reunion of such patients as are capable of enjoying society of an evening in the drawing-room, has a very cheering and salutary mental influence. There is a prejudice abroad that lunatics should not associate with lunatics; and that such mutual intercourse has a tendency to aggravate the disease. This is a fallacy. Nay, strange as it may appear, the delusions under which lunatics labour are sometimes corrected and dissipated by the sallies of wit and repartees of their fellow-patients. Thus, Pinel relates the case of a celebrated watchmaker in Paris, who became deranged from prosecuting the idea of perpetual motion. His derangement was characterized by this singularity—He believed that he had been guillotined, that his head had been mixed with some of the other victims, and the judges, repenting of their cruel verdict, ordered their heads to be replaced on their respective bodies. By some mistake, he conceived that the head of one of his unfortunate companions had been placed on his shoulders, and this idea haunted him night and day. One of his fellow-patients, a convalescent of a lively and jocular turn, one day directed his attention to the celebrated miracle of St. Denis, who carried his head under his arm and kissed it as he went along. Hereupon arose a discussion. The watchmaker insisting on the possibility of the fact, and citing his own case in support of it, upon which his companion burst out into a fit of laughter, and asked him how St. Denis could possibly contrive to kiss his own head? Was it with his heels? This unexpected repartee struck the lunatic forcibly; he became conscious of the absurdity of the idea; he retired, confused, amidst the laughter of all present, and never afterwards mentioned the misplacement of his head. There can be no doubt that observations of a striking nature, applied with tact, may sometimes have the effect of disabusing the insane mind of its illusions. Dr. Cox relates the case of a patient who asserted that he was the Holy Ghost. A gentleman present exclaimed, "You the Holy Ghost! What proof have you to adduce?"—"I know that I am," was his answer. The gentleman said, "How is this possible? there is but one Holy Ghost, is there? how, then, can you be the Holy Ghost, and I be so too?" He appeared surprised and puzzled, and after a short pause, said, "But are *you* the Holy Ghost?"—The other answered, "Did you not know that I was?" The patient replied, "I did not know it before! Why, then, I cannot be the Holy Ghost!" Thus, in lunatic asylums we have observed impressions arise out of incidental conversations which have at once awakened reflection, and a light has suddenly broken in upon the mind, which, "like a heavenly ray, has appeared to guide Reason back to her deserted throne."

Another important advantage arising from these establishments, is the clinical and pathological instruction derived from them; for the appointment of competent and well-educated medical men to reside in, and take charge of them, who consequently devote their entire attention to this department of science, enables them to acquire a knowledge of the phenomena of mental disease which is of the greatest importance. It is now well understood that insanity, particularly in its incipient stages, is as curable a disease as any other in the catalogue of human affliction; but experience is as clearly necessary for the treatment of this as for the treatment of any other malady. The medical practitioner, who has never handled a stethoscope, or explored the thoracic region, will have little success in the treatment of pneumonic affections; and he who has not made a speciality of the study of cerebral affections, and watched the clouding and unclouding of the mind in all its different phases of aberration, will have as little chance of success in the treatment of insanity. Hence, these institutions, both at home and abroad, have contributed in an eminent degree to the advancement of science, and the relief of suffering humanity.

When we review the history of public and private lunatic asylums, and compare their past with their present condition;—when we look to the various provisions, and the practical administration of the present act of Parliament, it appears manifest to us that the legislature has thrown every possible protection round the liberty of the subject; at the same time, that it has provided ample security for this unhappy class of sufferers being skilfully, kindly, and humanely treated;—and we feel assured that, when the evidence we have now referred to, is fairly and impartially considered, these establishments will be pronounced in every respect deserving the confidence of the public.

